# Weight-adjusted waist index is positively and linearly associated with all-cause and cardiovascular mortality in metabolic dysfunction-associated steatotic liver disease: findings from NHANES 1999-2018

**DOI:** 10.3389/fendo.2024.1457869

**Published:** 2024-09-30

**Authors:** Weijie Liu, Xiulin Yang, Ting Zhan, Min Huang, Xiaorong Tian, Xia Tian, Xiaodong Huang

**Affiliations:** ^1^ Department of Gastroenterology, Tongren Hospital of Wuhan University (WuHan Third Hospital), Wuhan, China; ^2^ Department of Gastroenterology, Zhongnan Hospital of Wuhan University, Wuhan, China

**Keywords:** metabolic dysfunction-associated steatotic liver disease, weight-adjusted waist index, mortality, body mass index, obesity

## Abstract

**Background:**

Metabolic dysfunction-associated steatotic liver disease (MASLD) is the most common chronic liver disease. Body mass index (BMI) is the most used obesity index but has important limitations. The weight-adjusted waist index (WWI) is a novel obesity metric and accurately reflects body composition. We explored the association of WWI with all-cause and cardiovascular disease (CVD) mortality in MASLD.

**Methods:**

Adult participants with MASLD were included from NHANES 1999-2018. WWI was calculated by dividing the waist circumference (WC) by the square root of body weight. MASLD was diagnosed by the presence of hepatic steatosis and at least one cardiometabolic risk factor in the absence of other causes of steatosis. A fatty liver index ≥60 suggested the presence of hepatic steatosis. Mortality data was obtained by prospectively linking to the National Death Index. Multivariate Cox proportional hazards regression analyses were used to explore these associations and multiple adjustment models were constructed including crude, partial, and fully adjusted models.

**Results:**

After adjusting for all covariates including BMI, WWI remained positively and linearly associated with all-cause and CVD mortality in MASLD (hazard ratios [HR] 1.247 and 1.218, respectively). Higher WWI was associated with a significantly increased risk of mortality (both p for trend <0.05). There was an “obesity paradox” between BMI and all-cause mortality in MASLD, with significantly lower all-cause mortality in those with overweight/obesity compared to normal BMI (HR 0.625 and 0.596, respectively, p for trend = 0.024), and no association between BMI and CVD mortality. Interaction analyses indicated that these associations were influenced by several demographic variables and disease status. Time-dependent receiver operating characteristic curves indicated that the predictive value of WWI for mortality in MASLD was higher than that of BMI, WC, and waist-to-height ratio across all follow-up durations.

**Conclusions:**

WWI was positively and linearly associated with all-cause and CVD mortality in MASLD, whereas BMI did not accurately reflect mortality risk. WWI provided the optimal predictive value for mortality compared to traditional obesity indicators. These findings emphasize the potential use of WWI as a novel obesity indicator for mortality risk assessment, stratification, and prevention in MASLD.

## 1 Introduction

Metabolic dysfunction-associated steatotic liver disease (MASLD), the new nomenclature for previous nonalcoholic fatty liver disease (NAFLD), is a clinicopathologic condition characterized by the presence of steatotic liver disease (SLD, i.e., hepatic steatosis) (and the exclusion of other causes of steatosis) accompanied by the presence of at least one cardiometabolic risk factor (CMRF) (1–3). MASLD is currently emerging as the most common chronic liver disease in the world, affecting more than one-third of adults (4). MASLD is now the leading cause of liver-related morbidity and mortality, and it is among the fastest growing cause of liver transplantation and hepatocellular carcinoma (5). In the U.S., MASLD causes an economic burden of approximately $100 billion annually and is expected to continue to grow in the future (5). In addition, MASLD is not limited to liver-related consequences, but is also strongly associated with the development of a variety of extrahepatic complications, including cardiovascular disease (CVD), metabolic disorders, chronic kidney disease (CKD), and multiple cancers (6).

Cumulative evidence suggests that MASLD is associated with significantly increased all-cause mortality (7, 8). Obesity is a major risk factor for the development of MASLD and affects the clinical outcomes of individuals, and weight loss through lifestyle interventions and bariatric surgery is considered the cornerstone of MASLD management (9). However, as a traditional indicator of obesity, body mass index (BMI) reflects only overall obesity and fails to capture visceral fat accumulation and differentiate body composition (10, 11). The “obesity paradox”, whereby BMI-defined obesity may be associated with improved clinical outcomes, has now been shown to exist in a variety of diseases (11). In fact, the so-called obesity paradox has been documented in NAFLD as well. Numerous longitudinal cohort studies have demonstrated that patients with lean NAFLD (BMI <25 kg/m^2^), although having fewer metabolic disturbances, nevertheless have significantly higher all-cause and/or CVD mortality than the non-lean NAFLD population, although inconsistent findings exist (12–15). A recent meta-analysis incorporating 7 cohort studies demonstrated a significant increase in all-cause mortality in lean NAFLD patients compared to non-lean NAFLD patients (15). Other meta-analyses have shown that liver-related mortality and CVD mortality may also be higher in lean NAFLD populations than in non-lean NAFLD individuals (13, 14), suggesting that there is a different clinical profile between lean and non-lean NAFLD populations and that BMI-defined obesity does not correctly reflect mortality risk in NAFLD populations. Although there is a lack of research to demonstrate whether lean MASLD populations have similar findings, given the limitations of BMI, the use of obesity metrics that more accurately reflect central obesity and body composition may be useful in the assessment and stratification of MASLD mortality risk.

To address this issue, Park et al. proposed a relatively novel indicator of obesity, the weight-adjusted waist index (WWI) (16). WWI has been shown to be linearly associated with cardiovascular and all-cause mortality in the general population and has better predictive value than traditional obesity parameters including BMI (16). Subsequent clinical studies have demonstrated that WWI accurately reflects body composition, including fat mass and muscle mass, in the general population (17, 18). In addition, as a reflection of central obesity, the WWI weakened the association with BMI, providing a more accurate reflection of the degree of obesity. However, whether WWI is associated with mortality in people with MASLD and independent of BMI remains unexplored. Here, we aimed to explore the association of WWI with all-cause and CVD mortality in individuals with MASLD by leveraging nationally representative data from the National Health and Nutrition Examination Survey (NHANES) and to reveal whether the BMI-defined obesity paradox exists in MASLD populations. These findings may indicate that WWI, as a simple and easily accessible anthropometric indicator, is independently associated with mortality in people with MASLD, and that WWI may have an important role in mortality risk assessment, stratification, and targeted prevention in individuals with MASLD.

## 2 Methods

### 2.1 Study design and population

NHANES is a major epidemiologic survey conducted by the National Center for Health Statistics (NCHS) to assess the health and nutritional status of community-dwelling populations in the U.S. Since 1999, the NHANES program has been updated every two years, and data are collected and evaluated on an ongoing basis. NHANES is a series of nationally representative population-based cross-sectional surveys characterized by a complex multistage probability sampling design. We followed the NHANES 1999-2018 MASLD population until December 31, 2019, and obtained mortality data prospectively; therefore, this study was a prospective cohort study. The NCHS Ethics Review Board (ERB) approved all NHANES protocols, and all participants provided written informed consent. NHANES is a publicly accessible database and therefore exempt from ethical approval at our institution.

**Figure 1:**
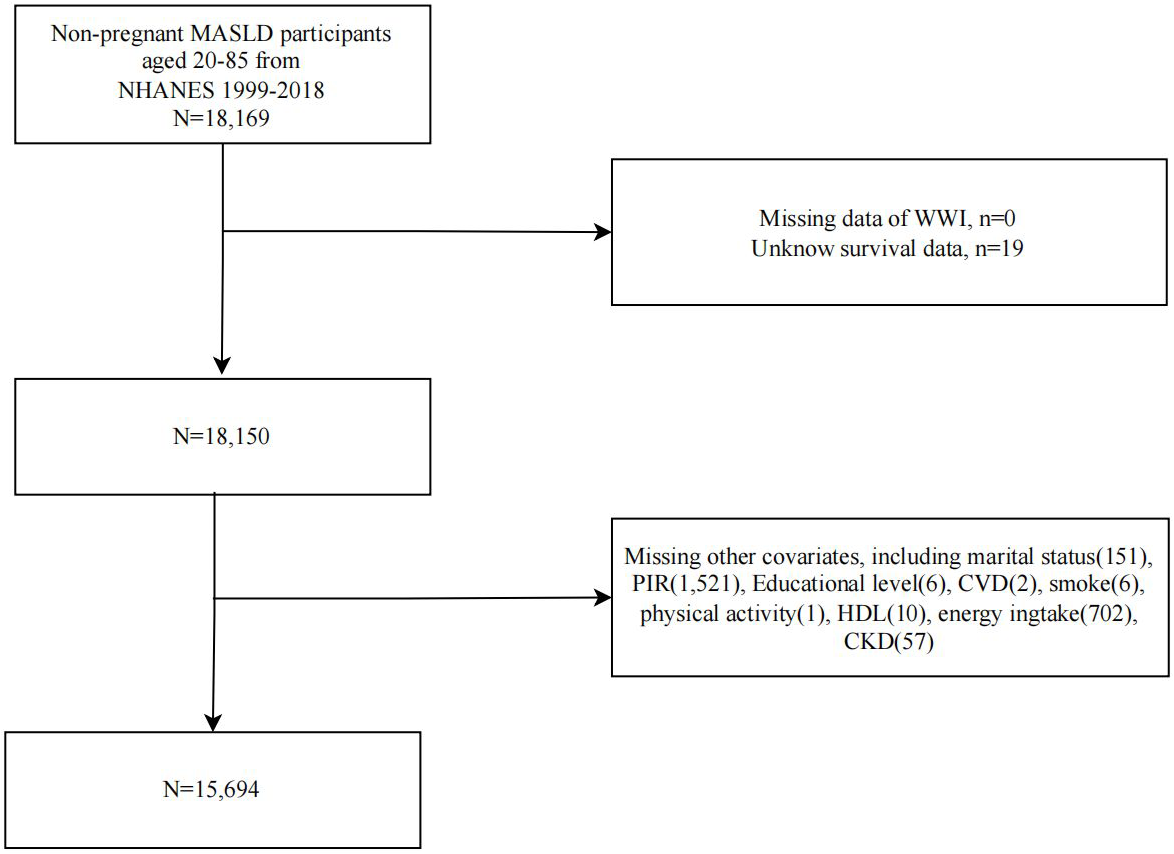
Flowchart of study population selection, NHANES 1999-2018.

A flowchart of study population selection and exclusion is shown in **Figure 1**. We first included 18,169 non-pregnant MASLD participants from NHANES 1999-2018 and sequentially excluded participants with missing WWI (n=0), survival data (n=19), and covariates (n=2456). Ultimately, 15,694 MASLD participants were included in further analyses.

### 2.2 Evaluation of WWI

According to the publication by Park et al (16), WWI was calculated based on an individual’s waist circumference (WC) (cm) divided by the square root of their body weight (kg). WC and body weight were collected by skilled medical personnel at the mobile examination center. The collected data is then recorded, quality controlled, and data processed to form the final document. In our study, WWI was the exposure variable and was treated as either a continuous variable (cm/√kg per unit) or a categorical variable (tertiles, T1-T3).

### 2.3 Diagnosis of MASLD

MASLD was defined as having at least 1 CMRF in addition to SLD and exclusion of other causes of steatosis (1). SLD refers to the presence of evidence of hepatic steatosis (lipid accumulation of more than 5% in hepatocytes) (1). CMRF was defined as one of the following five: BMI ≥25 kg/m^2^ or WC ≥94 cm (men)/≥80 cm (women), fasting blood glucose (FBG) ≥100 mg/dL/HbA1c ≥5.7%/history of type 2 diabetes (T2D)/on T2D therapy, blood pressure ≥130/85 mm Hg or on hypertension therapy, serum triglycerides (TG) ≥ 150 mg/dL or on lipid-lowering treatment, high-density lipoprotein cholesterol (HDL-C) <40 mg/dL (men)/<50 mg/dL (women) or on lipid-lowering therapy (1). FBG is defined as blood glucose measured the next morning before breakfast after an overnight 9h fast. We assessed hepatic steatosis using a widely validated noninvasive marker, the Fatty Liver Index (FLI), in which an FLI ≥60 indicated the presence of hepatic steatosis. The FLI is based on the calculation of BMI, WC, gamma-glutamyltransferase (GGT), and triglycerides (TG) with an accuracy of 0.84 (19). FLI is a noninvasive anthropometric and serologic-based diagnostic marker of hepatic steatosis calculated as follows: FLI = (e^0.953*ln (TG) + 0.139*BMI + 0.718*ln (GGT) + 0.053*WC - 15.745^)/(1 + e^0.953*ln (TG) + 0.139*BMI + 0.718*ln (GGT) + 0.053*WC - 15.745^) × 100 (19). In addition, to verify the consistency of the results, we used another widely used noninvasive marker of hepatic steatosis, the USFLI. The USFLI, the FLI developed by Ruhl et al. that is appropriate for the U.S. population, was calculated using the formula: USFLI = (e^-0.8073 × non-Hispanic black + 0.3458 × Mexican American + 0.0093 × age + 0.6151 × ln (GGT) + 0.0249 × WC + 1.1792 × ln (insulin) + 0.8242 × ln (glucose) -14.7812^)/(1 + e ^−0.8073 × non-Hispanic black + 0.3458 × Mexican American + 0.0093 × age + 0.6151 × ln (GGT) + 0.0249 × WC + 1.1792 × ln (insulin) + 0.8242 × ln (glucose) -14.7812^) × 100 (20). A USFLI ≥ 30 was considered to have hepatic steatosis (20). The USFLI is developed based on NHANES, which has a diagnostic AUC of 0.80 based on age, race/ethnicity, WC, GGT, fasting insulin, and FBG levels (20).

### 2.4 Mortality information collection

We obtained mortality data in the MASLD population through December 31, 2019, through prospective matching with the publicly accessible National Death Index database. CVD mortality was extracted from codes related to deaths from cardiac and cerebrovascular diseases, including I00-I09, I11, I13, I20-I51, and I60-I69.

### 2.5 Covariates

We included age, sex, race/ethnicity (categorized as non-Hispanic White/non-Hispanic Black/Mexican American/Other Hispanic/Other race), educational attainment (categorized as <high school/high school/greater than high school education level), household income-poverty ratio (PIR), marital status, smoking, physical activity, dietary energy intake, diabetes, hypertension, CVD, CKD, TG, total cholesterol (TC), HDL-C, and BMI. Smoking was categorized according to participant responses on the SMQ questionnaire (21) as never smokers (less than 100 cigarettes in their lifetime), former smokers (at least 100 cigarettes in their lifetime but quit in the past 12 months), and current smokers (at least 100 cigarettes in their lifetime and currently smoking). Physical activity was categorized as no/moderate/vigorous physical activity participation based on self-report on the PAQ questionnaire. Total dietary energy intake was obtained from the 24-h dietary recall interview (kcal/d) (22). The presence of diabetes was suggested by one of the following: self-reported history of diabetes, FBG ≥7.0 mmol/L, HbA1c ≥6.5%, random blood glucose ≥11.1 mmol/L, or use of antidiabetic medications (23). Hypertension was diagnosed based on a history of hypertension, a blood pressure test ≥140/90 mmHg, or antihypertensive medication use (24). CVD history (including angina/coronary heart disease/congestive heart failure/stroke/heart attack) was obtained based on self-report in the MCQ questionnaire (25). CKD was defined as a urine albumin/creatinine ratio (uACR) ≥ 30 mg/g and/or an estimated glomerular filtration rate (eGFR) < 60 ml/min/1.73 m^2^ (26). Serum lipid profile data were obtained through the NHANES biochemical tests file.

### 2.6 Statistical analysis

All analyses in this study were appropriately weighted according to NHANES analytic guidelines given the complex study design of NHANES (27). All analyses were performed using EmpowerStats software and R version 4.2.3, and a two-sided P value of less than 0.05 was considered statistically significant. In the baseline analysis, we grouped the baseline MASLD population according to the tertile distribution of the WWI. Between-group differences for continuous variables (mean ± standard error) and categorical variables (number [percentage]) were tested using weighted ANOVA and chi-square analyses, respectively. Kaplan-Meier (KM) survival curves were used to assess differences in time-dependent survival probabilities in MASLD populations across WWI tertiles and to examine between-group differences using the log-rank test. Multivariate Cox proportional hazards regression models were used to explore the independent association of WWI with mortality in the MASLD population. Crude models did not adjust for any covariates, model 1 partially adjusted for age, sex, race/ethnicity, education, PIR, and marital status, model 2 adjusted additionally for smoking, physical activity, dietary energy intake, diabetes mellitus, hypertension, CVD, CKD, TG, TC, and HDL-C based on model 1, and model 3 continued to adjust for BMI based on model 2. In addition, we also explored the association of BMI with mortality in the MASLD population, with the variables adjusted for by the individual models being broadly consistent with the WWI (except for adjusted for the WWI in Model 3). Restricted cubic spline (RCS) modeling was used to explore potential nonlinear or dose-response associations and select appropriate nodes for smooth curve fitting. Stratified analyses were used to explore whether these associations remained stable across subgroups and to explore potential effect modifiers using interaction analyses. In sensitivity analyses, we used the USFLI to diagnose MASLD to verify the reliability of the findings. Receiver operating characteristic (ROC) curves were used to assess the predictive value of the WWI and other traditional obesity metrics for mortality in the MASLD population and to assess discriminatory power by area under the curve (AUC). Differences in AUC between WWI and other obesity indicators were performed using the DeLong test (28).

## 3 Results

### 3.1 Baseline characteristics

We included 15,694 MASLD participants, with a mean age of 49.757 years, and 52.707% were male and 69.271% were non-Hispanic White. The tertiles of WWI were taken as T1 (<11.1 cm/√kg), T2 (11.1-11.8 cm/√kg), and T3 (≥11.8 cm/√kg). As WWI tertiles increased, participants were older; had lower PIR, energy intake, TG, and TC; had higher BMI, WC, WHtR, and HDL-C; and were more likely to be female, non-Hispanic White, single, ≤high school educated, former smokers, physically uninvolved participants, and have diabetes, hypertension, CKD, and CVD (**Table 1**).

**Table 1:** Baseline analysis of the MASLD population according to WWI tertiles, NHANES 1999-2018.

Variables	Total (n=15,694)	T1 (n=5232)	T2 (n=5232)	T3 (n=5230)	P value
**BMI, kg/m^2^**	34.127 ± 0.085	32.481 ± 0.105	34.062 ± 0.119	36.377 ± 0.148	<0.0001
**WWI**	11.386 ± 0.009	10.694 ± 0.006	11.446 ± 0.003	12.237 ± 0.007	<0.0001
**WC, cm**	112.091 ± 0.169	105.375 ± 0.174	112.639 ± 0.204	120.379 ± 0.291	<0.0001
**WHtR**	0.664 ± 0.001	0.608 ± 0.001	0.666 ± 0.001	0.735 ± 0.002	<0.0001
**Age, year**	49.757 ± 0.221	43.456 ± 0.253	50.788 ± 0.301	56.963 ± 0.363	<0.0001
**PIR**	2.918 ± 0.031	3.176 ± 0.037	2.942 ± 0.037	2.551 ± 0.041	<0.0001
**Energy intake, kcal/day**	2162.251 ± 10.471	2362.362 ± 17.586	2144.876 ± 19.338	1916.469 ± 15.743	<0.0001
**TG, mg/dL**	205.217 ± 1.830	213.298 ± 3.243	203.606 ± 2.961	196.292 ± 3.182	<0.001
**TC, mg/dL**	201.527 ± 0.559	203.929 ± 0.737	202.273 ± 0.928	197.524 ± 0.975	<0.0001
**HDL-C, mmol/L**	1.180 ± 0.004	1.151 ± 0.005	1.175 ± 0.007	1.223 ± 0.006	<0.0001
**Sex**					<0.0001
male	8071 (52.707)	3475 (67.163)	2762 (53.630)	1834 (32.552)	
female	7623 (47.293)	1757 (32.837)	2470 (46.370)	3396 (67.448)	
**Race**					<0.0001
Mexican American	3229 (9.519)	908 (8.683)	1164 (10.502)	1157 (9.542)	
Non-Hispanic Black	3130 (10.434)	1305 (12.088)	1026 (10.218)	799 (8.483)	
Non-Hispanic White	7068 (69.271)	2230 (67.782)	2277 (68.439)	2561 (72.161)	
Other Hispanic	1347 (5.617)	384 (5.208)	490 (6.270)	473 (5.440)	
Other Race	920 (5.158)	405 (6.239)	275 (4.571)	240 (4.375)	
**Marital Status**					<0.0001
non-single	9933 (67.411)	3519 (71.095)	3481 (70.038)	2933 (59.638)	
single	5761 (32.589)	1713 (28.905)	1751 (29.962)	2297 (40.362)	
**Education**					<0.0001
<high school	2048 (6.272)	357 (3.491)	678 (6.504)	1013 (9.698)	
high school	6251 (38.390)	1957 (34.849)	2138 (39.649)	2156 (41.689)	
>high school	7395 (55.338)	2918 (61.660)	2416 (53.847)	2061 (48.613)	
**Smoking**					<0.0001
never	8318 (52.870)	2963 (57.788)	2664 (49.077)	2691 (50.541)	
former	4554 (28.976)	1233 (24.138)	1615 (31.507)	1706 (32.590)	
now	2822 (18.154)	1036 (18.074)	953 (19.416)	833 (16.869)	
**Physical activity**					<0.0001
no	8391 (47.903)	2352 (40.234)	2797 (49.124)	3242 (56.711)	
moderate	3870 (27.446)	1273 (27.010)	1325 (27.329)	1272 (28.151)	
vigorous	3433 (24.651)	1607 (32.756)	1110 (23.547)	716 (15.137)	
**CVD**					<0.0001
no	13444 (88.075)	4871 (94.250)	4500 (88.014)	4073 (79.968)	
yes	2250 (11.925)	361 (5.750)	732 (11.986)	1157 (20.032)	
**Diabetes**					<0.0001
no	11350 (77.790)	4482 (88.963)	3787 (78.021)	3081 (62.744)	
yes	4344 (22.210)	750 (11.037)	1445 (21.979)	2149 (37.256)	
**Hypertension**					<0.0001
no	7179 (49.635)	3048 (60.654)	2347 (48.012)	1784 (36.836)	
yes	8515 (50.365)	2184 (39.346)	2885 (51.988)	3446 (63.164)	
**CKD**					<0.0001
no	12203 (82.306)	4595 (90.621)	4072 (82.176)	3536 (71.441)	
yes	3491 (17.694)	637 (9.379)	1160 (17.824)	1694 (28.559)	

Between-group differences for continuous variables (mean ± standard error) and categorical variables (number [percentage]) were tested using weighted ANOVA and chi-square analyses, respectively.

### 3.2 Association of WWI with all-cause and CVD mortality in MASLD

**Figure 2:**
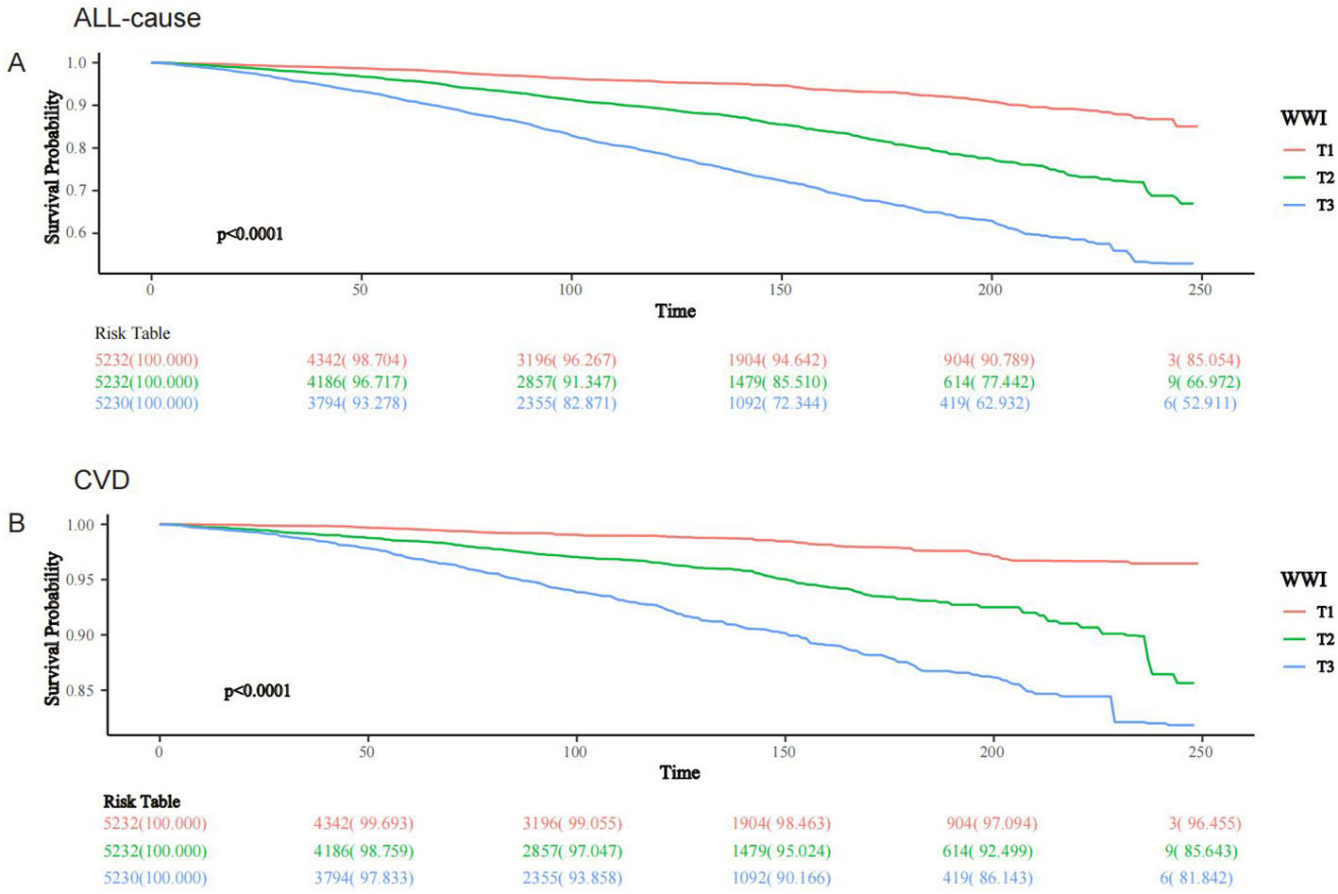
KM survival analysis of WWI and all-cause and CVD mortality in people with MASLD, NHANES 1999-2018. **(A)** All-cause; **(B)** CVD.

After a median follow-up duration of 108 months (interquartile range = 56-159 months), 2462 (11.81%) individuals with MASLD died, with 818 CVD-related deaths. The KM survival curves showed that both all-cause and CVD-related survival probabilities over time were significantly lower in the MASLD population as the WWI tertile increased (both log-rank test p < 0.0001) (**Figure 2**). In the crude model, Model 1, and Model 2, WWI was positively associated with all-cause mortality in the MASLD population. After adjusting for all confounders including BMI, WWI remained positively associated with all-cause mortality (hazard ratio [HR] = 1.247, 95% confidence interval [CI] = 1.137-1.369, p < 0.0001). Compared to T1, WWI at both T2 and T3 was associated with significantly increased all-cause mortality (HR 1.273 and 1.437, respectively, p for trend <0.0001). Similarly, in Model 3, WWI was positively associated with CVD mortality in the MASLD population (HR=1.218, 95% CI=1.026-1.447, p=0.024). Higher WWI was associated with significantly increased CVD mortality (p for trend = 0.032) (**Table 2**). After adjusting for all confounders, BMI (continuous) was not significantly associated with all-cause mortality in the MASLD population (HR=1.004, 95% CI=0.993-1.015, p=0.507). Interestingly, all-cause mortality was significantly lower in both MASLD individuals with overweight and obesity status compared to those with normal BMI (HR 0.625 and 0.596, respectively, p for trend = 0.024), suggesting the existence of an “obesity paradox” (**Supplementary Table S1**). However, BMI was not significantly associated with CVD mortality in people with MASLD, regardless of whether BMI was regarded as a continuous or categorical variable (**Supplementary Table S2**).

**Table 2:** Association of WWI with all-cause and CVD mortality in the MASLD population, NHANES 1999-2018.

	Crude Model HR (95%CI) P-value	Model 1 HR (95%CI) P-value	Model 2 HR (95%CI) P-value	Model 3 HR (95%CI) P-value
All-cause				
**WWI**	2.468 (2.287,2.664) <0.0001	1.425 (1.304,1.558) <0.0001	1.257 (1.148,1.376) <0.0001	**1.247 (1.137,1.369)** **<0.0001**
WWI tertiles				
**T1**	ref	ref	ref	ref
**T2**	2.582 (2.183,3.055) <0.0001	1.415 (1.211,1.652) <0.0001	1.283 (1.099,1.497) 0.002	**1.273 (1.091,1.486)** **0.002**
**T3**	5.051 (4.408,5.788) <0.0001	1.791 (1.539,2.083) <0.0001	1.459 (1.257,1.694) <0.0001	**1.437 (1.236,1.671)** **<0.0001**
**P for trend**	<0.0001	<0.0001	<0.0001	**<0.0001**
CVD-cause				
**WWI**	2.622 (2.339,2.938) <0.0001	1.448 (1.230,1.704) <0.0001	1.259 (1.059,1.497) 0.009	**1.218 (1.026,1.447)** **0.024**
WWI tertiles				
**T1**	ref	ref	ref	ref
**T2**	3.122 (2.391,4.077) <0.0001	1.536 (1.168,2.019) 0.002	1.385 (1.045,1.835) 0.024	**1.344 (1.015,1.779)** **0.039**
**T3**	6.042 (4.666,7.825) <0.0001	1.855 (1.354,2.542) <0.001	1.483 (1.074,2.048) 0.017	**1.395 (1.012,1.921)** **0.042**
	<0.0001	<0.001	0.024	**0.032**

Crude models did not adjust for any covariates, model 1 partially adjusted for age, sex, race/ethnicity, education, PIR, and marital status, model 2 adjusted additionally for smoking, physical activity, dietary energy intake, diabetes mellitus, hypertension, CVD, CKD, TG, TC, and HDL-C based on model 1, and model 3 continued to adjust for BMI based on model 2. The bolded portion suggests that the fully adjusted results are statistically significant.

### 3.3 RCS analysis

**Figure 3:**
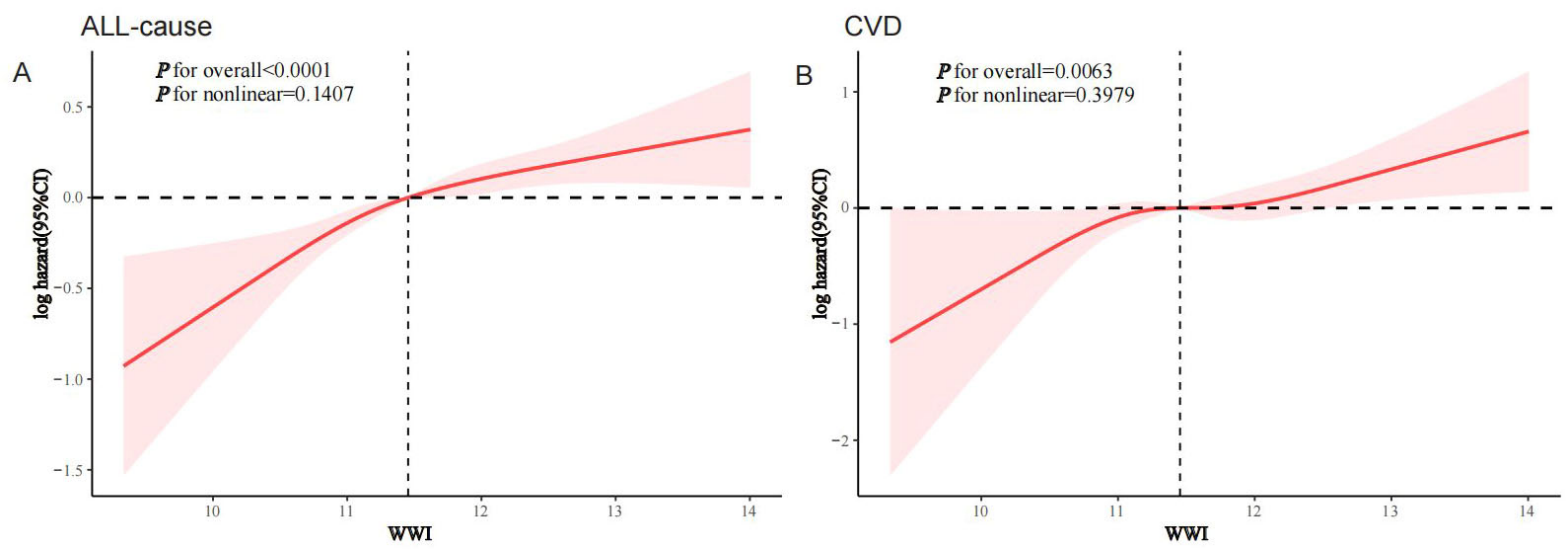
RCS analysis of the association of WWI with all-cause and CVD mortality in the MASLD population, NHANES 1999-2018. **(A)** All-cause; **(B)** CVD.

RCS modeling demonstrated a linear association between WWI and both all-cause and CVD mortality in the MASLD population (p for nonlinearity was 0.1407 and 0.3979, respectively), suggesting a dose-response association (**Figure 3**).

### 3.4 Stratified analysis

**Figure 4:**
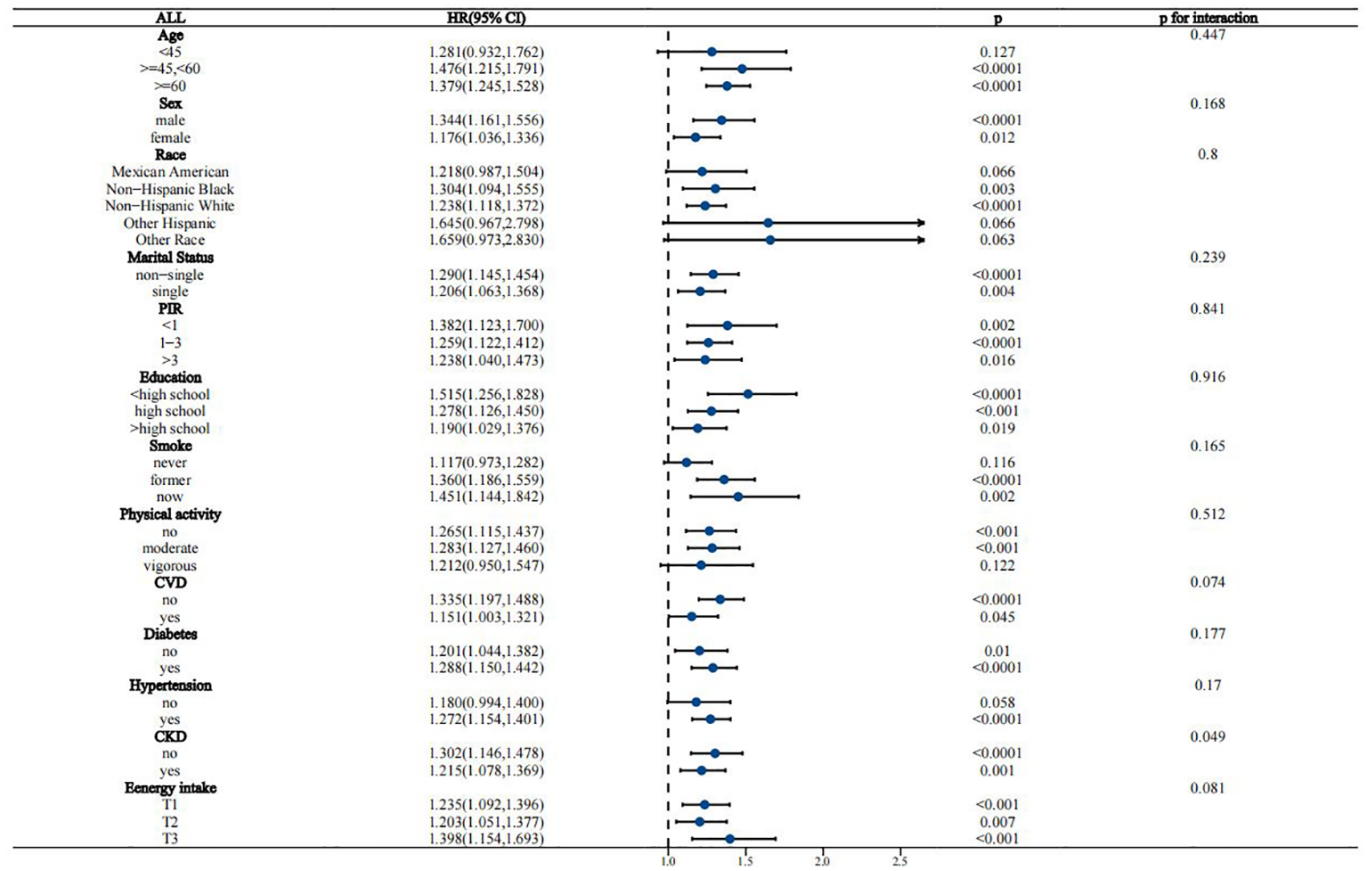
Stratified analysis of the association between WWI and all-cause mortality in the MASLD population.

**Figure 5:**
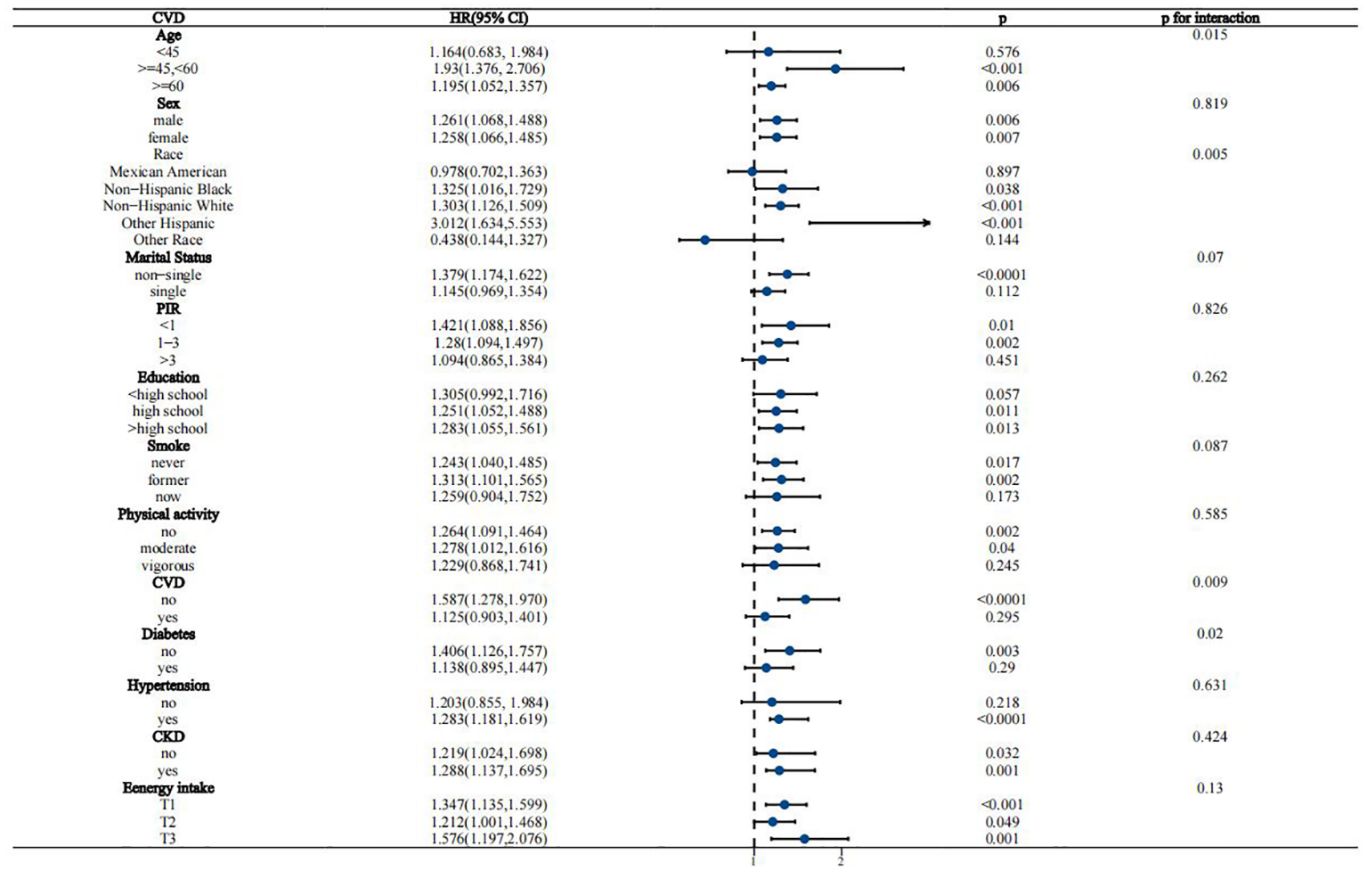
Stratified analysis of the association between WWI and CVD mortality in the MASLD population.

Interaction analyses suggested that the association between WWI and all-cause mortality in the MASLD population remained stable across most subgroups, except for CKD, which borderline significantly affected this association (p for interaction = 0.049) (**Figure 4**). For the association of WWI with CVD mortality, age, race/ethnicity, CVD history, and diabetes status were identified as significant effect modifiers (p for interaction 0.015, 0.005, 0.009, and 0.02, respectively) (**Figure 5**). This association was more pronounced in people aged 45-60 years, other Hispanic ethnic groups, people with no history of CVD, and people without diabetes.

### 3.5 Sensitivity analysis

The diagnosis of MASLD using USFLI yielded broadly similar findings. After adjusting for all covariates, the WWI remained associated with all-cause and CVD mortality in the MASLD population (HR 1.259 and 1.202, respectively) (**Supplementary Tables S3**, **S4**). Similarly, the association of BMI with USFLI-MASLD was not significantly altered. All-cause mortality was significantly lower in MASLD populations with overweight and obesity than in those with normal BMI (HR 0.616 and 0.581, respectively, p for trend = 0.028), whereas BMI was not associated with CVD mortality in MASLD populations (**Supplementary Tables S5**, **S6**).

### 3.6 ROC analysis

**Figure 6:**
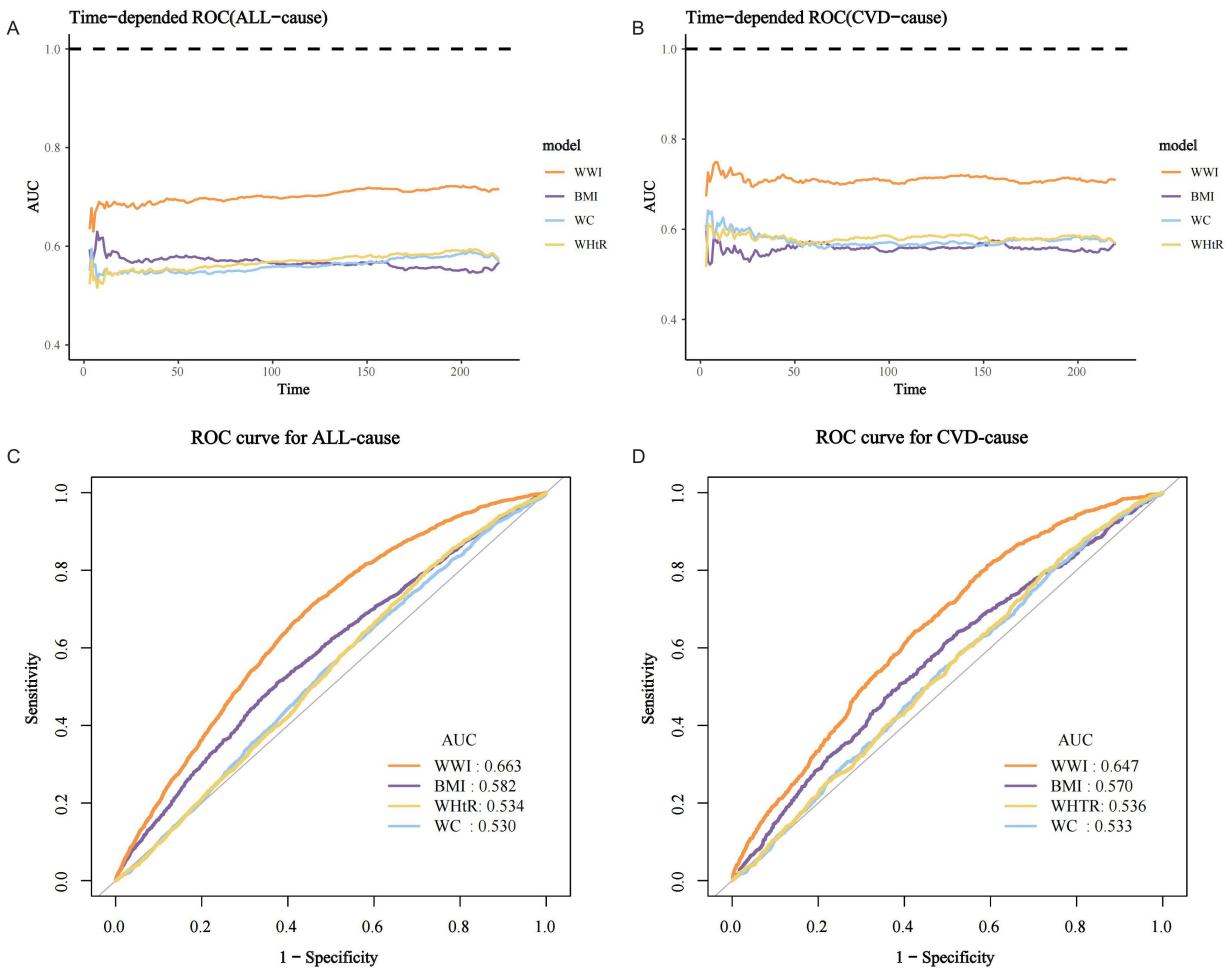
ROC curves for mortality prediction in the MASLD population by the WWI and other traditional obesity indicators. **(A)** Time-dependent ROC for all-cause mortality; **(B)** Time-dependent ROC for CVD mortality; **(C)** ROC for all-cause mortality at 75% follow-up duration; **(D)** ROC for CVD mortality at 75% follow-up duration.

Time-dependent ROC curves demonstrated that the predictive power (AUC) of WWI for both all-cause and CVD mortality in the MASLD population was significantly higher than that of BMI, WC, and waist-to-height ratio (WHtR) across all follow-up time periods (**Figures 6A, B**). For example, the AUC value for the prediction of all-cause mortality in the MASLD population for the WWI at 75% follow-up length was 0.663, which was significantly higher than BMI (AUC=0.582), WHtR (AUC=0.534), and WC (AUC=0.530) (**Figure 6C**). Similarly, the AUC value for the prediction of CVD mortality by WWI was 0.647, which was significantly higher than BMI (AUC=0.570), WHtR (AUC=0.536), and WC (AUC=0.533) (**Figure 6D**). Specific details of the ROC curves were presented in **Table 3**. The WWI had significantly higher AUC values for the prediction of mortality than other traditional obesity metrics (p for all AUC comparisons <0.0001). The best cutoff values for WWI to predict all-cause and CVD mortality in the MASLD population were 11.4600 and 11.2627, respectively.

**Table 3:** Details of ROC curves for WWI and other obesity indicators for mortality prediction in the MASLD population.

Test	AUC	95%CI low	95%CI upper	Best threshold	Specificity	Sensitivity	Comparison (p-value)
All-cause							
**WWI**	0.6631	0.6521	0.6741	11.4600	0.5444	0.7096	Ref.
**BMI**	0.5819	0.5696	0.5943	31.4950	0.6250	0.5097	**<0.0001**
**WC**	0.5305	0.5184	0.5425	109.5500	0.5141	0.5431	**<0.0001**
**WHTR**	0.5344	0.5226	0.5462	0.6131	0.2717	0.8054	**<0.0001**
CVD							
**WWI**	0.6472	0.6292	0.6651	11.2627	0.4062	0.8130	Ref.
**BMI**	0.5698	0.5495	0.5901	32.8850	0.4994	0.6186	**<0.0001**
**WC**	0.5331	0.5136	0.5527	108.4500	0.4686	0.5892	**<0.0001**
**WHTR**	0.5358	0.5165	0.5551	0.6154	0.2751	0.7971	**<0.0001**

AUC, area under the curve; WWI, weight-adjusted waist index; BMI, body mass index; WC, waist circumference; WHtR, waist-to-height ratio. The bolded portion suggests that the fully adjusted results are statistically significant.

## 4 Discussions

In this nationwide prospective cohort study, our findings suggest that a relatively novel anthropometric indicator of obesity, WWI, is positively and linearly associated with all-cause and CVD mortality in the MASLD population. Higher WWI was associated with significantly increased all-cause and CVD mortality. Interestingly, there is an “obesity paradox” with respect to BMI for all-cause mortality. All-cause mortality was significantly lower in the overweight/obese population compared to the normal BMI population. However, BMI was not significantly associated with CVD mortality in the MASLD population. These associations are influenced by several factors, including CKD, age, race/ethnicity, CVD, and diabetes. Importantly, our study demonstrated that the predictive power of the WWI for all-cause and CVD mortality in the MASLD population was significantly higher than that of other traditional obesity metrics across all follow-up time periods. Overall, our findings suggest that WWI may serve as the optimal obesity-indicator-related prognostic factor in people with MASLD, whereas existing BMI-defined obesity does not accurately reflect mortality risk in people with MASLD.

The WWI is an index used to reflect central obesity, combining the strength of WC by standardizing it to body weight, while weakening the association with BMI. As an indicator of obesity, the WWI directly and specifically assesses central obesity with stability, reliability, and applicability to different races and ethnicities (29). To the best of our knowledge, there are no real-world studies demonstrating an association between WWI and mortality in MASLD or NAFLD populations. Several studies have shown that WWI is significantly associated with the prevalence of NAFLD in the general population. Using data from NHANES 2017-2020, Shen et al. indicated that WWI was associated with hepatic steatosis as assessed by controlled attenuation parameters in the general U.S. adult population (30). Another study using NHANES 2017-2020 similarly suggested that WWI was significantly and positively associated with the development of both NAFLD and liver fibrosis in the general population (31). A retrospective cohort study demonstrated that each unit of increased WWI was associated with a 72% increased risk of NAFLD, especially in non-obese men (32). In addition, there is growing evidence that WWI is strongly associated with the risk of mortality in the general population. A prospective cohort study using NHANES 2005-2014 demonstrated that higher WWI (≥11.33) was associated with a significantly increased risk of all-cause (HR=1.68) and CVD mortality (HR=1.95) in the general US adult population (33). Using a prospective cohort from China, Ding et al. showed that WWI in the highest quartile (≥11.2) was associated with significantly increased all-cause (HR=1.36, 95% CI=1.17-1.58) and CVD mortality (HR=1.43, 95% CI=1.15-1.77) (34). Another prospective cohort study from China similarly demonstrated that WWI was significantly associated with all-cause mortality in the general elderly population (HR=2.66 in the highest tertile) and that its predictive value for mortality was superior to BMI, WC, and WHtR (35). Finally, a prospective cohort study using NHANES 2011-2018 suggested that WWI was positively associated with all-cause mortality in the non-Asian general population and had similarly superior predictive power (WWI: AUC = 0.697; BMI: AUC = 0.524; WC: AUC = 0.562) (36). Our study demonstrated for the first time that WWI is positively associated with both all-cause and CVD mortality in MASLD populations, currently the most common chronic liver disease, providing new insights into the mortality prediction, stratification, and potential preventive value of WWI in MASLD. Consistent with previous studies, the mortality predictive ability of WWI was significantly superior to traditional obesity metrics, including BMI, WC, and WHtR, suggesting that it may be useful as a new simple and easily accessible anthropometric indicator to replace BMI or be used conjointly for mortality risk assessment in individuals with MASLD. As a relatively new and not internationally accepted indicator of obesity, the WWI may have important clinical relevance. Future studies are needed to further explore whether WWI can be used for mortality prediction in MASLD populations in clinical practice and whether it adds to the discriminatory power of traditional prediction models.

Accumulating evidence suggests that WWI may accurately reflect body composition, which is positively correlated with fat mass and negatively correlated with muscle mass. Kim et al. utilized a national cross-sectional study to show that WWI was positively associated with total body fat percentage and negatively associated with appendicular lean mass (ALM)/body weight and bone mineral density (18). WWI can be perceived as an integrative indicator of body composition, and a higher WWI reflects poor body composition. Another cross-sectional analysis from Korea similarly demonstrated that WWI was positively associated with total abdominal fat area, visceral fat area, and total tissue fat percentage, and negatively associated with ALM and ALM/height squared (17). Results from the Multi-Ethnic Study of Atherosclerosis similarly revealed that WWI was positively associated with abdominal fat mass and negatively associated with abdominal muscle mass, both cross-sectionally and longitudinally (37). Emerging clinical evidence suggests that WWI is associated with sarcopenia or sarcopenic obesity (SO) in the general population or in specific populations. A recent cross-sectional analysis from NHANES indicated that WWI was significantly and positively associated with sarcopenia in the general population (men: OR=14.55; women: OR=2.86) (38). Another cross-sectional analysis from Korea demonstrated that WWI had the best predictive value for SO in the elderly population compared to other obesity indicators and was only present in men (AUC=0.784) (39). Similarly, WWI has the best predictive value for SO in the T2D population (40). Overall, WWI, a novel anthropometric indicator, is a more accurate reflection of body composition than other obesity indicators, with higher WWI representing increased adipose tissue (especially abdominal/visceral fat) mass and decreased muscle mass. Thus, the poor body composition represented by higher WWI may explain the positive association with mortality in the MASLD population. Notably, the prevalence of sarcopenia/SO is significantly higher in the MASLD/NAFLD population than in the general population and is associated with a significantly increased risk of mortality (41). Sarcopenia and MASLD/associated fibrosis share common pathogenic mechanisms, including systemic low-grade chronic inflammation, insulin resistance, oxidative stress, hyperammonemia, abnormal myokine secretion, and other pathway disturbances (42). An important mechanism by which sarcopenia increases mortality in the MASLD population may be that sarcopenia is associated with progression of fibrosis in the MASLD/NAFLD population, and fibrosis staging is the most potent prognostic factor in the MASLD population (43). In addition, WWI may serve as an indicator of central obesity and is associated with important pathophysiologic hallmarks in MASLD including adipose tissue dysfunction and abnormal gene expression in lipid metabolism (44), which may partially explain the effect of WWI on mortality in MASLD.

Notably, interaction analyses revealed several potential effect modifiers, including CKD, age, race/ethnicity, CVD, and diabetes. The effect of WWI on mortality risk in the MASLD population was more pronounced in those without CKD, CVD, and diabetes. These conditions all have important crosstalk with MASLD and significantly contribute to excess mortality in MASLD (45). The presence of these diseases may partially explain the increased mortality in MASLD and may diminish the effect of WWI on mortality risk. Thus, our findings suggest the need to focus on the association and predictive value of WWI with mortality risk in populations without these diseases. In addition, our results suggest that the association between WWI and CVD mortality is more pronounced in specific age- and race/ethnicity-specific populations, suggesting the need to consider the impact of specific demographic characteristics on the association between WWI and CVD mortality in MASLD populations.

In addition, apart from WWI, other plasma-derived markers may also have an important role in risk assessment and stratification of all-cause/CVD mortality in MASLD populations, such as Sirtuin 1. The anti-aging gene Sirtuin 1 is important to the prevention of age-related diseases such as obesity, CVD and MASLD (46). Sirtuin 1 inactivation leads to multiple organ disease syndrome that involves the adipose tissue, heart, liver, pancreas and brain (47, 48). Specific racial groups and communities may be sensitive to the downregulation of Sirtuin 1 and may have an important role in CVD mortality risk assessment in individuals with MASLD (49). Previous evidence suggests that Sirtuin 1 may act as a key gene in the prevention of obesity (50). Sirtuin 1 is thought to be involved in the regulation of hepatic lipid metabolism, aging of the liver, and cardiovascular risk in MASLD (51, 52), and thus may be associated with disease severity and prognosis. Therefore, early plasma measurement of Sirtuin 1 in life may accurately reflect CVD mortality risk in people with MASLD. Although due to the limitations of the NHANES data, we were unable to explore whether plasma Sirtuin 1 is a more sensitive marker of CVD mortality in the MASLD population. As an important pathophysiologic participant in MASLD, Sirtuin 1 may become a more sensitive, accurate, and clinically useful marker for CVD mortality assessment in MASLD populations in the future. Further studies are needed to explore whether Sirtuin 1 serves as a more sensitive and accurate marker for CVD mortality risk assessment in MASLD populations and whether it can be incorporated into mortality risk prediction models for MASLD populations together with WWI.

Our study has some significant strengths. It was a prospective cohort study and took full account of potential confounders to minimize study bias and ensure the reliability of the conclusions. NHANES is a nationally representative survey with a large sample and multiple races/ethnicities, making the results generalizable. However, there are limitations to our study. First, the diagnosis of MASLD was based on noninvasive markers rather than imaging tools, which may lack accuracy. Imaging tools for hepatic steatosis assessment were lacking in NHANES 1999-2018 (transient elastography to detect steatosis was only introduced in 2017-2018). However, both FLI and USFLI are widely validated noninvasive markers with good accuracy and have yielded broadly consistent findings in sensitivity analyses, demonstrating the stability of our results. Second, given that current risk assessment models for mortality in MASLD generally consist of multiple important risk factors, we did not explore here the predictive value of WWI along with other risk factors for mortality in MASLD. Our results suggest that WWI has the optimal predictive value compared with traditional obesity metrics, which provides new insights for subsequent predictive model construction. Future studies are needed to confirm whether WWI has additional value for traditional mortality risk prediction models.

## 5 Conclusions

In a large national prospective cohort study, WWI was positively and linearly associated with all-cause and CVD mortality in people with MASLD. There was an “obesity paradox” between BMI and all-cause mortality in MASLD, with significantly lower mortality in those with overweight/obesity. These associations were influenced by some demographic variables and some diseases. The WWI had the optimal predictive value for all-cause and CVD mortality in MASLD compared with other traditional obesity metrics across all follow-up time periods. These findings emphasize the predictive, stratification, and potential preventive value of WWI for mortality risk in MASLD populations. Our study suggested that WWI accurately reflected the risk of mortality in MASLD rather than BMI. Future research needs to explore whether WWI provides additional value to traditional prognostic models.

## Data Availability

Publicly available datasets were analyzed in this study. This data can be found here: This study analyzed publicly available datasets and can be found at https://www.cdc.gov/nchs/nhanes/.
