# Supplementary material for: Weight-adjusted waist index is positively and linearly associated with all-cause and cardiovascular mortality in metabolic dysfunction-associated steatotic liver disease: findings from NHANES 1999-2018
Source: Front Endocrinol (Lausanne). 2024 Sep 30;15:1457869. doi: 10.3389/fendo.2024.1457869 (PMC11471496; doi:10.3389/fendo.2024.1457869)
Supplement: Supplementary file 1 [file DataSheet1.docx]

**Table S1**. Association of BMI with all-cause mortality in the MASLD population.

| **All-cause** | **Crude Model**  **HR (95%CI)** | **P-value** | **Model 1**  **HR (95%CI)** | **P-value** | **Model 2**  **HR (95%CI)** | **P-value** | **Model 3**  **HR (95%CI)** | **P-value** |
| --- | --- | --- | --- | --- | --- | --- | --- | --- |
| **BMI** | 0.977(0.967,0.988) | <0.0001 | 1.018(1.007,1.028) | <0.001 | 1.010(0.999,1.021) | 0.089 | 1.004 (0.993,1.015) | 0.507 |
| **BMI** |  |  |  |  |  |  |  |  |
| **<25 kg/m^2^** | ref | ref | ref | ref | ref | ref | ref | ref |
| **25-30 kg/m^2^** | 0.490(0.338,0.710) | <0.001 | 0.541(0.372,0.787) | 0.001 | 0.638(0.444,0.914) | 0.014 | **0.625(0.436,0.897)** | **0.011** |
| **≥30 kg/m^2^** | 0.380(0.264,0.549) | <0.0001 | 0.592(0.410,0.854) | 0.005 | 0.639(0.443,0.924) | 0.017 | **0.596(0.414,0.857)** | **0.005** |
| **P for trend** |  | <0.0001 |  | 0.727 |  | 0.186 |  | **0.024** |

Crude models did not adjust for any covariates, model 1 partially adjusted for age, sex, race/ethnicity, education, PIR, and marital status, model 2 adjusted additionally for smoking, physical activity, dietary energy intake, diabetes mellitus, hypertension, CVD, CKD, TG, TC, and HDL-C based on model 1, and model 3 continued to adjust for WWI based on model 2.

**Table S2**. Association of BMI with CVD mortality in the MASLD population.

| **CVD** | **Crude Model**  **HR (95%CI)** | **P-value** | **Model 1**  **HR (95%CI)** | **P-value** | **Model 2**  **HR (95%CI)** | **P-value** | **Model 3**  **HR (95%CI)** | **P-value** |
| --- | --- | --- | --- | --- | --- | --- | --- | --- |
| **BMI** | 0.984(0.966,1.002) | 0.087 | 1.035(1.016,1.055) | <0.001 | 1.022(1.001,1.044) | 0.041 | 1.017(0.996,1.039) | 0.118 |
| **BMI** |  |  |  |  |  |  |  |  |
| **<25 kg/m^2^** | ref | ref | ref | ref | ref | ref | ref | ref |
| **25-30 kg/m^2^** | 0.896(0.445,1.804) | 0.758 | 0.973(0.468,2.023) | 0.942 | 1.143(0.540,2.418) | 0.726 | 1.123(0.530,2.382) | 0.762 |
| **≥30 kg/m^2^** | 0.708(0.350,1.431) | 0.336 | 1.159(0.561,2.395) | 0.689 | 1.188(0.559,2.526) | 0.654 | 1.114(0.523,2.371) | 0.78 |
| **P for trend** |  | 0.012 |  | 0.088 |  | 0.595 |  | 0.939 |

Crude models did not adjust for any covariates, model 1 partially adjusted for age, sex, race/ethnicity, education, PIR, and marital status, model 2 adjusted additionally for smoking, physical activity, dietary energy intake, diabetes mellitus, hypertension, CVD, CKD, TG, TC, and HDL-C based on model 1, and model 3 continued to adjust for WWI based on model 2.

**Table S3**. Association of WWI with all-cause mortality in the USFLI-MASLD population.

| **All-cause** | **Crude Model**  **HR (95%CI)** | **P-value** | **Model 1**  **HR (95%CI)** | **P-value** | **Model 2**  **HR (95%CI)** | **P-value** | **Model 3**  **HR (95%CI)** | **P-value** |
| --- | --- | --- | --- | --- | --- | --- | --- | --- |
| **WWI** | 2.450(2.253,2.665) | <0.0001 | 1.443(1.300,1.602) | <0.0001 | 1.274(1.147,1.415) | <0.0001 | **1.259(1.131,1.401)** | **<0.0001** |
| **WWI tertiles** |  |  |  |  |  |  |  |  |
| T1 | ref | <0.0001 | ref | ref | ref | ref | ref | ref |
| T2 | 2.590(2.173,3.088) | <0.0001 | 1.407(1.189,1.665) | <0.0001 | 1.270(1.076,1.500) | 0.005 | **1.255(1.063,1.482)** | **0.007** |
| T3 | 4.956(4.266,5.757) | <0.0001 | 1.798(1.504,2.151) | <0.0001 | 1.464(1.232,1.740) | <0.0001 | **1.429(1.203,1.698)** | **<0.0001** |
| **P for trend** | <0.0001 | | <0.0001 | | <0.0001 | | **<0.0001** | |

Crude models did not adjust for any covariates, model 1 partially adjusted for age, sex, race/ethnicity, education, PIR, and marital status, model 2 adjusted additionally for smoking, physical activity, dietary energy intake, diabetes mellitus, hypertension, CVD, CKD, TG, TC, and HDL-C based on model 1, and model 3 continued to adjust for BMI based on model 2.

**Table S4**. Association of WWI with CVD mortality in the USFLI-MASLD population.

| **CVD** | **Crude Model**  **HR (95%CI)** | **P-value** | **Model 1**  **HR (95%CI)** | **P-value** | **Model 2**  **HR (95%CI)** | **P-value** | **Model 3**  **HR (95%CI)** | **P-value** |
| --- | --- | --- | --- | --- | --- | --- | --- | --- |
| **WWI** | 2.568(2.272,2.903) | <0.0001 | 1.420(1.182,1.707) | <0.001 | 1.249(1.033,1.510) | 0.022 | **1.202(1.002,1.450)** | **0.047** |
| **WWI tertiles** |  |  |  |  |  |  |  |  |
| T1 | ref | ref | ref | ref | ref | ref | ref | ref |
| T2 | 3.556(2.633,4.802) | <0.0001 | 1.713(1.246,2.356) | <0.001 | 1.575(1.145,2.166) | 0.005 | **1.520(1.108,2.086)** | **0.009** |
| T3 | 6.041(4.470,8.162) | <0.0001 | 1.859(1.274,2.713) | 0.001 | 1.518(1.044,2.206) | 0.029 | 1.413(0.977,2.045) | 0.067 |
| **P for trend** |  | <0.0001 |  | 0.002 |  | 0.064 |  | 0.148 |

Crude models did not adjust for any covariates, model 1 partially adjusted for age, sex, race/ethnicity, education, PIR, and marital status, model 2 adjusted additionally for smoking, physical activity, dietary energy intake, diabetes mellitus, hypertension, CVD, CKD, TG, TC, and HDL-C based on model 1, and model 3 continued to adjust for BMI based on model 2.

**Table S5**. Association of BMI with all-cause mortality in the USFLI-MASLD population.

| **ALL** | **Crude Model**  **HR (95%CI)** | **P-value** | **Model 1**  **HR (95%CI)** | **P-value** | **Model 2**  **HR (95%CI)** | **P-value** | **Model 3**  **HR (95%CI)** | **P-value** |
| --- | --- | --- | --- | --- | --- | --- | --- | --- |
| **BMI** | 0.978(0.968,0.989) | <0.0001 | 1.020(1.009,1.031) | <0.001 | 1.012(1.000,1.024) | 0.051 | 1.006(0.994,1.018) | 0.352 |
| **BMI** |  |  |  |  |  |  |  |  |
| **<25 kg/m^2^** | ref | ref | ref | ref | ref | ref | ref | ref |
| **25-30 kg/m^2^** | 0.485(0.324,0.726) | <0.001 | 0.528(0.350,0.796) | 0.002 | 0.621(0.417,0.925) | 0.019 | **0.616(0.416,0.912)** | **0.016** |
| **≥30 kg/m^2^** | 0.368(0.246,0.552) | <0.0001 | 0.578(0.383,0.873) | 0.009 | 0.620(0.411,0.936) | 0.023 | **0.581(0.388,0.869)** | **0.008** |
| **P for trend** | <0.0001 | | 0.716 | | 0.186 | | **0.028** | |

Crude models did not adjust for any covariates, model 1 partially adjusted for age, sex, race/ethnicity, education, PIR, and marital status, model 2 adjusted additionally for smoking, physical activity, dietary energy intake, diabetes mellitus, hypertension, CVD, CKD, TG, TC, and HDL-C based on model 1, and model 3 continued to adjust for WWI based on model 2.

**Table S6**. Association of BMI with CVD mortality in the USFLI-MASLD population.

| **CVD** | **Crude Model**  **HR (95%CI)** | **P-value** | **Model 1**  **HR (95%CI)** | **P-value** | **Model 2**  **HR (95%CI)** | **P-value** | **Model 3**  **HR (95%CI)** | **P-value** |
| --- | --- | --- | --- | --- | --- | --- | --- | --- |
| **BMI** | 0.984(0.966,1.002) | 0.085 | 1.036(1.016,1.056) | <0.001 | 1.024(1.002,1.047) | 0.031 | 1.019(0.997,1.042) | 0.083 |
| **BMI** |  |  |  |  |  |  |  |  |
| **<25 kg/m^2^** | ref | ref | ref | ref | ref | ref | ref | ref |
| **25-30 kg/m^2^** | 0.985(0.474,2.047) | 0.967 | 1.058(0.489,2.288) | 0.886 | 1.232(0.563,2.697) | 0.602 | 1.225(0.559,2.684) | 0.612 |
| **≥30 kg/m^2^** | 0.773(0.370,1.616) | 0.494 | 1.286(0.595,2.777) | 0.522 | 1.305(0.592,2.876) | 0.509 | 1.241(0.563,2.733) | 0.592 |
| **P for trend** |  | 0.015 |  | 0.047 |  | 0.438 |  | 0.712 |

Crude models did not adjust for any covariates, model 1 partially adjusted for age, sex, race/ethnicity, education, PIR, and marital status, model 2 adjusted additionally for smoking, physical activity, dietary energy intake, diabetes mellitus, hypertension, CVD, CKD, TG, TC, and HDL-C based on model 1, and model 3 continued to adjust for WWI based on model 2.

SUPPLEMENTARY MATERIAL: R code for the adjustment models for the association of WWI and BMI with all-cause and cardiovascular mortality in the MASLD population (note: ‘wwi-masld.csv’ is our cleaned raw data table).

#Setting the work path

setwd('D:///RRR')

#Loading required libraries

library(nhanesR)

library(reshape2)

library(survival)

library(rms)

library(survey)

#Read file, identify categorical variables and convert to categorical variable format

d<-read.csv(file = 'wwi-masld.csv')

d$agec<-as.factor(d$agec)

d$sex<-as.factor(d$sex)

d$PIRC<-as.factor(d$PIRC)

d$en<-as.factor(d$en)

d$race<-as.factor(d$race)

d$education<-as.factor(d$education)

d$race<-as.factor(d$race)

d$marital<-as.factor(d$marital)

d$sex<-as.factor(d$sex)

d$marital<-as.factor(d$marital)

d$activity<-as.factor(d$activity)

d$smoke<-as.factor(d$smoke)

#WWI Trifecta

quantiles<-quantile(d$wwi, probs = c(1/3,2/3))

d$c <- cut(d$wwi, breaks = c(-Inf, quantiles, Inf),

labels = c("T1", "T2", "T3"), include.lowest = TRUE)

#Variables needed to calculate P for trend

d$cc<-as.numeric(d$c)

d$bmic<-as.factor(d$bmic)

#Variables needed to calculate P for trend

d$bmicc<-as.numeric(d$bmic)

#The data is weighted and a matrix is generated

nhs<-svy_design(data = d)

#COX regression for calculating all-cause mortality

#crude model

svy_uv.cox(design = nhs,time = 'time',status = 'all',x = c('wwi','c','cc'),round = 3)

#model 1

svy_uv.cox(design = nhs,time = 'time',status = 'all',x = c('wwi','c','cc'),round = 3,adjust = c('age','sex','race','marital','PIR','education'))

#model 2

svy_uv.cox(design = nhs,time = 'time',status = 'all',x = c('wwi','c','cc'),round = 3,adjust = c('age','sex','race','marital','PIR','education','smoke','activity','DM','Hypertension','energy','CVD','tg','HDL','CKD','tc'))

#model 3

svy_uv.cox(design = nhs,time = 'time',status = 'all',x = c('wwi','c','cc'),round = 3,adjust = c('age','sex','race','marital','PIR','education','smoke','activity','DM','Hypertension','energy','CVD','tg','HDL','CKD','tc','bmi'))

#crude model

svy_uv.cox(design = nhs,time = 'time',status = 'all',x = c('bmi','bmic','bmicc'),round = 3)

#model 1

svy_uv.cox(design = nhs,time = 'time',status = 'all',x = c('bmi','bmic','bmicc'),round = 3,adjust = c('age','sex','race','marital','PIR','education'))

#model 2

svy_uv.cox(design = nhs,time = 'time',status = 'all',x = c('bmi','bmic','bmicc'),round = 3,adjust = c('age','sex','race','marital','PIR','education','smoke','activity','DM','Hypertension','energy','CVD','tg','HDL','CKD','tc'))

#model 3

svy_uv.cox(design = nhs,time = 'time',status = 'all',x = c('bmi','bmic','bmicc'),round = 3,adjust = c('age','sex','race','marital','PIR','education','smoke','activity','DM','Hypertension','energy','CVD','tg','HDL','CKD','tc','wwi'))

#COX regression for calculating CVD-cause mortality

#crude model

svy_uv.cox(design = nhs,time = 'time',status = 'cvd',x = c('wwi','c','cc'),round = 3)

#model 1

svy_uv.cox(design = nhs,time = 'time',status = 'cvd',x = c('wwi','c','cc'),round = 3,adjust = c('age','sex','race','marital','PIR','education'))

#model 2

svy_uv.cox(design = nhs,time = 'time',status = 'cvd',x = c('wwi','c','cc'),round = 3,adjust = c('age','sex','race','marital','PIR','education','smoke','activity','DM','Hypertension','energy','CVD','tg','HDL','CKD','tc'))

#model 3

svy_uv.cox(design = nhs,time = 'time',status = 'cvd',x = c('wwi','c','cc'),round = 3,adjust = c('age','sex','race','marital','PIR','education','smoke','activity','DM','Hypertension','energy','CVD','tg','HDL','CKD','tc','bmi'))

#crude model

svy_uv.cox(design = nhs,time = 'time',status = 'cvd',x = c('bmi','bmic','bmicc'),round = 3)

#model 1

svy_uv.cox(design = nhs,time = 'time',status = 'cvd',x = c('bmi','bmic','bmicc'),round = 3,adjust = c('age','sex','race','marital','PIR','education'))

#model 2

svy_uv.cox(design = nhs,time = 'time',status = 'cvd',x = c('bmi','bmic','bmicc'),round = 3,adjust = c('age','sex','race','marital','PIR','education','smoke','activity','DM','Hypertension','energy','CVD','tg','HDL','CKD','tc'))

#model 3

svy_uv.cox(design = nhs,time = 'time',status = 'cvd',x = c('bmi','bmic','bmicc'),round = 3,adjust = c('age','sex','race','marital','PIR','education','smoke','activity','DM','Hypertension','energy','CVD','tg','HDL','CKD','tc','wwi'))
